# Factors associated with injury and re-injury occurrence in female pole dancers

**DOI:** 10.1038/s41598-021-04000-5

**Published:** 2022-01-07

**Authors:** Andrzej Szopa, Małgorzata Domagalska-Szopa, Aleksandra Urbańska, Monika Grygorowicz

**Affiliations:** 1 Department of Physiotherapy, School of Health Sciences in Katowice, Medical University of Silesia in Katowice, Medyków 12, 40-752 Katowice, Poland; 2 Department of Medical Rehabilitation, School of Health Sciences in Katowice, Medical University of Silesia in Katowice, Medyków 12, 40-752 Katowice, Poland; 3 Rehabilitace Poliklinika, Sady Komenského 605/1, 737 01, Czech Cieszyn, Czech Republic; 4 Department of Physiotherapy, Poznan University of Medical Sciences, Fredry 10, 61-701 Poznań, Poland; 5 Rehasport Clinic FIFA Medical Centre of Excellence, Górecka 30, 60-201 Poznań, Poland

**Keywords:** Risk factors, Epidemiology

## Abstract

The aim of the study was to recognise what participant-, training- and post-injury-related factors are associated with an injury and re-injury occurrence in female pole dancers (PDs). 320 female PDs fulfilled a custom survey. 1050 injuries were reported by 276 PDs, 59% of injuries were related to lower extremity, 39% to upper extremity and 10% to spine and trunk. 156 PDs reported sustaining a re-injury, and overall, 628 re-injuries were reported. The median weekly pole-specific training session volume was 90 min and 240 min in the low and high qualified group, respectively. The total training volume was 180 min in the low qualified PDs and 240 min in the high qualified group. PDs with higher height and spending more time on pole-specific training in studio and on other forms of training have higher odds of sustaining an injury. PDs with lower level of experience in training, who sustained an injury, and who had a shorter pause between the moment of injury and the return to performance, and thus who did not fully recover, have higher odds of sustaining a re-injury. Sport-specific injury prevention strategies should be developed and implemented in this cohort, since over 85% of pole dancers reported sustaining some kind of injury.

## Introduction

Since the recreational pole dance (PD) schools progressively emphasize their main function as a community for providing a fitness activity^1^, the popularity of the PD as a form of aerobic exercises^2^ has increased among women of all ages around the world over the last few decades^3,4^. Consequently, PD is now recognized as a sport discipline that combines elements of dance and acrobatics^5,6^, and for which national and international championships are organized^7^ (starting from the first World Championship, held in Amsterdam in 2005^4^, and for which the International Pole Sports Federation (IPSF) took another step towards making the sport an Olympic one^8^. It has been confirmed that advanced-level participation in PD classes, completed for ≥ 30 min on ≥ five days per week (total ≥ 150 min), might fulfil the American College of Sports Medicine guidelines for improving health and cardiorespiratory fitness^9^.

However, apart from health positive aspects of pole dance, data also shows that PD specific manoeuvres conducted during classes (coded by the IPSF into cupid, handspring, front split-down pole, cradle spin, and extended butterfly^9^) can provoke substantial loading to the wrists and shoulders, which might lead to overloading the musculoskeletal system^6^. Sometimes, especially when the pole dancer has to perform some specific manoeuvres based on controlled drops or dives along the pole, the overload forces equal to 2–3 G^10^. Previous studies have assessed injury location and analysed the prevalence of PD injuries and the association among injuries and different factors^6,11^. Pole dancers reported sustaining the most common injuries in the shoulder and wrist, and in majority cases the injuries were acutely sustained^11^. Sprains, strains are typical for these athletes, but more severe injuries such as concussions and fractures are also common as a result of falls from the pole^12^, with reports of even serious injuries requiring surgery, e.g. dislocated clavicle fracture or isolated transverse process fracture^7^. Although a few recent papers examined physical and physiological demands of PD^9,10^, there is still very little knowledge about the relation between the training-related factors and the occurrence of injuries. To our knowledge, no scientific paper was published regarding the association among post-injury situation and the occurrence of re-injury. Thus, the aim of the study was to recognise what participant-related, training-related, and post-injury-related factors are associated with an injury and re-injury occurrence in a cohort of female pole dancers, and to calculate a logistic model most adequately describing these relations.

## Material and methods

The study was designed as cross-sectional survey with retrospective collection of all necessary information. Between March and June 2019, fifty pole dance schools (recognized by the Polish Pole Sports Community associated in the IPSF) from 12 out of all 16 provinces in Poland were invited to the study and its representatives were asked to distribute the online questionnaire among female pole dancers. The survey consisted of two sections with 13 and 12 questions respectively, grouped according to the following aspects: (71) demographic data and pole dancer profile including: age, height, weight, BMI, school name, previous injuries not related with pole dance); (2) PD training-specific and sport participation data related to: the level of experience, weekly participation volume, additional activities; and (3) injury history i.e. type, location, severity of injury expressed as time-loss data. The participants were informed that the questionnaire served for research purposes and the authors were interested in analysing the factors that are related with injury and re-injury occurrence in this specific population. Informed consent was obtained from all the participants in the study, and all responders declared their voluntary participation in the study. The Bioethical Committee of Silesian Medical University provided ethical approval of the study (no. PCN/CBN/0022/KB/145/19). The experiments in the present study were performed in accordance with the Helsinki Declaration.

Assuming a 95% confidence level and a 5% margin of error, which is common in epidemiological research, a sample size of 317 was required, in a population of 1800 female pole dancers in Poland.

Any physical complaint sustained by a participant that resulted from a pole dance training session, irrespective of the need for medical attention or time lost from pole dance activities was classified as an injury. Injuries while doing additional activities were not counted. Additionally, we classified an injury as a re-injury only when it was followed by a previous injury to the same place in the body.

To meet the criteria for this study, a participant had to be an active pole dancer for at least 6 months, and she had to be registered in one of the official PD schools in Poland. Moreover, the injury was confirmed by the medical examination, as was the return to full recovery—also was confirmed by the physician. Only positive marking of these two pieces of information allowed us to include a participant's results in further analysis. Missing one of these excluded a person from the study. No other inclusion or exclusion criteria were applied.

To investigate what factors are associated with an injury and re-injury occurrence in a cohort of female pole dancers we used a logistic regression with „injury occurrence” as dichotomous response variable (0 = no injuries, 1 = one or more injuries). As the explanatory variables (independent variables) we used several modifiable and non-modifiable factors. They were grouped into three categories: participant-, training-, and variables related to post-injury situation. The main participant-related explanatory variables investigated were: age in years (factor #1), weight in kilograms (factor #2), height in centimetres (factor #3) (all of these factors were qualified as continuous type of measures), abnormality of Body Mass Index of the pole dancer (factor #4), dichotomously categorized as 0 = normal or healthy weight (when BMI is ranged 18.5–24.9), and 1 = abnormal weight (underweight when BMI < 18.5 or overweight when BMI >  = 25), and information about any previous injury sustained by the pole dancer before regular participation in PD training dichotomously categorized as 0 = no and 1 = yes (factor #5). The training-related explanatory variables included the information about the role and experience of the pole dancers. They were dichotomously categorized as 0 = participant of the classes and 1 = instructor for the PD classes (factor #6), level of experience in PD training, 0 = beginner or basic level and 1 = advanced or extreme level (factor #7), information about any items used by pole dancer for her protection classified as 0 = no and 1 = yes (factor #8). Additional variables, which were considered as continuous measures were total months of training experience in pole dance, expressed in months (factor #9), pole-specific training performed in the dance studio expressed in minutes per week (factor #10), similarly calculated total volume of pole-specific training performed in the dance studio and additional physical activities, e.g. swimming, riding a bike, running (factor #11), and minutes dedicated for the warm-up before the training session (factor #12). For those pole dancers who sustained an injury, the participant- and training-related factors mentioned above were also used to assess the relative contributions for sustaining any re-injury (0 = no re-injury, 1 = re-injury reported). Additionally, 4 more explanatory variables dedicated to post-injury situation were added to the logistic regression model: dichotomous information whether the physiotherapy was applied after sustaining an injury or not, 0 = no, 1 = yes (factor #13), information whether the pole dancer had a pause from the training session or not, 0 = no, 1 = yes (factor #14), information whether the pole dancer sustained severe injury or not, 0 = no, 1 = yes (> 28 days of time loss in training) (factor #15) and information whether the pole dancer fully recovered, 0 = no, 1 = yes (factor #16). To evaluate the associations of all explanatory variables with injury and re-injury, two models of logistic regression analyses were performed and odds ratios with 95% confidence intervals (CI) were calculated. Variable selection for logistic regression models was based on backward stepwise empirical approach removing non-significant variables using p < 0.05 as the cut-off level. We started with a basic model containing all the predictors and then by removing the weakest contributor in every single model we finally developed the model containing only the variables significantly relevant for the injury or re-injury occurrence. Akaike Information Criterion (AIC) was calculated to observe the difference between the models analysed in backward stepwise, and the best subset models were specified among them, based on the minimum value of AIC. The calculations were performed using the PQStat 1.6.2. statistical program (PQStat Software, Poznan, Poland). A p-value of less than 0.05 was considered to be indicative of statistical significance.

## Results

500 questionnaires were distributed, 120 questionnaires were not completed. In 60 of them, it was not indicated that the injuries were verified by a physician. Therefore, those 60 were also excluded from the study. A total of 320 female pole dancers completed the questionnaire. Eighty-six percent of them (n = 276; mean ± SD (95%CI) age = 26 ± 5.3 (25.4–26.6) years, weight = 57.2 ± 6.9 (56.3–58) kg, height = 166.4 ± 5.6 (165.8–167.1) cm, BMI = 20.6 ± 2.2 (20.4–20.9) reported injury occurrence during their sport career, whereas almost 14% (n = 44; age = 26 ± 4.7 (24.6–27.4) years, weight = 57.8 ± 5.8 (56–59.5) kg, height = 165.8 ± 4.9 (164.3–167.2) cm, BMI = 21 ± 1.8 (20.5–21.6) did not report sustaining any injury. Over 48% (n = 156; age = 25.5 ± 4.9 (24.7–26.3) years, weight = 57.1 ± 7.1(56–58.3) kg, height = 166.4 ± 5.4 (165.6–167.3) cm, BMI = 20.6 ± 2.3 (20.3–21) of respondents confirmed sustaining a re-injury (second, or third time), whereas over 37% n = 120; age = 26.7 ± 5.8 (25.6–27.7) years, weight = 57.2 ± 6.7 (56–58.4) kg, height = 166.4 ± 5.8 (165.3–167.4) cm, BMI = 20.6 ± 2 (20.3–21) of all pole dancers sustained only one injury.

Thirty-three percent of respondents (n = 107) were categorised as highly qualified (representing advanced or extreme level of training experience). Twelve percent of all respondents (n = 39) had the highest training qualifications (PD instructors), and 67% (n = 213) had lower qualifications (beginners or basic level). The median weekly pole-specific training session volume was 90 (range 70–150) and 240 (range 120–450) minutes in low and high qualified group respectively. The total training volume (when additional activities e.g., swimming, running were taken into account) increased to 180 (range 120–240) minutes in low qualified dancers and 240 (range 120–300) minutes in the high qualified group.

A total of 1050 unique pole dance-related injuries were reported by 276 dancers. The majority of injuries were related to lower extremity (59%) and upper extremity (39%), with the lower percentage of spine and trunk injuries (10%). Muscles and tendons were injured most frequently (25%), followed by joints and ligaments (23%). The most common type of injury was contusion (60%) and tear (26%). One hundred fifty-six pole dancers reported sustaining a re-injury of different type and location, and overall, 628 re-injuries of the same place in the body were reported. One hundred and twenty respondents declared a total number of 422 injuries of different type and location, however none of them were followed by a previous injury to the same place in the body, thus none of them were classified as a re-injury.

In the analysed sample of pole dancers, injury occurrence was significantly associated with increased height and higher volume of training activity (both pole-specific training performed in the dance studio and additional training activity) (Table 1). The odds of reporting a re-injury increased significantly in pole dancers with shorter post-injury pause time, who did not fully recover and who declared a lower level of qualification (beginners or basic level) (Table 1). A detailed arrangement of predictors is presented in Table 2 with the best-fitting models that contain one or more explanatory variable. Table 2 also shows the best models selected for injury and re-injury occurrence respectively, depending on the AIC value.

**Table 1:** Detailed estimates from the logistic regression models of the injury and re-injury occurrence.

Model	Factors	No	Variable	β	SE	Wald	*P*	OR (95%CI)
Injury occurrence [y/n]	Participant’s-related factors	#1	Age [yrs]	0.01	0.04	0.03	0.8734	1.01 (0.94–1.08)
		#2	Weight [kg]	−0.05	0.03	2.46	0.1171	0.95 (0.89–1.01)
		#3	**Height [cm]**	**0.09**	**0.04**	**4.08**	**0.0433**	**1.09 (1.01–1.19)**
		#4	Abnormality of BMI [y/n]	0.64	0.54	1.44	0.2305	1.9 (0.66–5.45)
		#5	Previous non pole dance related injury [y/n]	0.5	0.37	1.8	0.1798	1.65 (0.79–3.43)
	Training-related factors	#6	Instructor [y/n]	−1.92	1.34	2.05	0.1523	0.15 (0.01–2.03)
		#7	Extreme level of training [y/n]	0.53	0.66	0.64	0.4253	1.7 (0.46–6.22)
		#8	Protection [y/n]	0.68	0.79	0.73	0.3916	1.97 (0.42–9.36)
		#9	Training [mnths]	0.04	0.03	2.05	0.1524	1.04 (0.98–1.11)
		#10	**Studio training [min/week]**	**0.01**	**0**	**3.9**	**0.0482**	**1.01 (1.01–1.02)**
		#11	**Total training [min/week]**	**0.01**	**0**	**8.3**	**0.0430**	**1.01 (1.01–1.02)**
		#12	Warm up [min]	0.02	0.04	0.22	0.6378	1.02 (0.94–1.11)
Re-injury occurrence [y/n]	Participant’s-related factors	#1	Age [yrs]	−0.03	0.03	1.23	0.2673	0.97 (0.92–1.02)
		#2	Weight [kg]	0.02	0.02	0.66	0.4157	1.02 (0.97–1.07)
		#3	Height [cm]	−0.02	0.03	0.51	0.4739	0.98 (0.93–1.04)
		#4	Abnormality of BMI [y/n]	0.36	0.35	1.06	0.3022	1.44 (0.72–2.86)
		#5	Previous non pole dance related injury [y/n]	−0.09	0.27	0.12	0.7241	0.91 (0.54–1.54)
	Training-related factors	#6	Instructor [y/n]	0.26	0.61	0.18	0.6688	1.3 (0.39–4.25)
		#7	**Extreme level of training [y/n]**	**−1.01**	**0.39**	**6.73**	**0.0095**	**0.37 (0.17–0.78)**
		#8	Protection [y/n]	0.09	0.42	0.05	0.8236	1.1 (0.48–2.51)
		#9	Training [mnths]	0.01	0.02	0.27	0.6002	1.01 (0.97–1.05)
		#10	Studio training [min/week]	0	0	0.03	0.8721	1 (1–1)
		#11	Total training [min/week]	0	0	0.09	0.7666	1 (1–1)
		#12	Warm up [min]	−0.02	0.02	0.64	0.422	0.98 (0.94–1.03)
	Post-injury-related factors	#13	Physiotherapy [y/n]	−0.18	0.44	0.16	0.6854	0.84 (0.36–1.97)
		#14	**Pause in training [y/n]**	**−0.75**	**0.33**	**5.16**	**0.0231**	**0.47 (0.25–0.9)**
		#15	Severe injury [y/n]	−0.28	0.43	0.43	0.5097	0.75 (0.32–1.75)
		#16	**Full recovery [y/n]**	**−0.94**	**0.39**	**5.85**	**0.0156**	**0.39 (0.18–0.84)**

SE, standard error; OR, odds ratio; CI, confidence interval.

**Table 2:** Best models identified for injury occurrence and for re-injury depending on AIC criterion.

Model	Model covariates for injury occurrence	AIC	No. of significant factors
1	#1,#2,#3,#4,#5,#6,#7,#8,#9,#10,#11,#12	232,07	#3,#10,#11
2	#2,#3,#4,#5,#6,#7,#8,#9,#10,#11,#12	230,09	#3,#10,#11
3	#2,#3,#4,#5,#6,#7,#8,#9,#10,#11	228,33	#3,#10,#11
4	#2,#3,#4,#5,#6,#8,#9,#10,#11	226,98	#3,#10,#11
5	#2,#3,#4,#5,#6,#9,#10,#11	226,03	#3,#9,#10,#11
6	**#2,#3,#5,#6,#9,#10,#11**	**225,44**	**#3,#9,#10,#11**
7	#2,#3,#6,#9,#10,#11	225,91	#3,#9,#10,#11
8	#2,#3,#9,#10,#11	225,48	#3,#10,#11
9	#2,#3,#10,#11	227,75*	#10,#11
10	#3,#10,#11	227,34	#10,#11
11	#10,#11	227,15	#10,#11
12	#11	240,14*	#11
	Model covariates for re-injury		
1	#1,#2,#3,#4,#5,#6,#7,#8,#9,#10,#11,#12,#13,#14,#15,#16	377.36	#7,#14,#16
2	#1,#2,#3,#4,#5,#6,#7,#8,#9,#11,#12,#13,#14,#15,#16	375.38	#7,#14,#16
3	#1,#2,#3,#4,#5,#6,#7,#9,#11,#12,#13,#14,#15,#16	373.43	#7,#14,#16
4	#1,#2,#3,#4,#5,#6,#7,#9,#12,#13,#14,#15,#16	371.50	#7,#14,#16
5	#1,#2,#3,#4,#6,#7,#9,#12,#13,#14,#15,#16	369.62	#7,#14,#16
6	#1,#2,#3,#4,#6,#7,#9,#12,#14,#15,#16	367.75	#7,#14,#16
7	#1,#2,#3,#4,#7,#9,#12,#14,#15,#16	366.11	#7,#14,#16
8	#1,#2,#3,#4,#7,#12,#14,#15,#16	364.75	#7,#14,#16
9	#1,#2,#3,#4,#7,#12,#14,#16	363.48	#7,#14,#16
10	#1,#2,#4,#7,#12,#14,#16	362.05	#7,#14,#16
11	#1,#2,#4,#7,#14,#16	360.88	#7,#14,#16
12	#1,#4,#7,#14,#16	359.18	#7,#14,#16
13	#1,#7,#14,#16	358.13	#7,#14,#16
14	**#7,#14,#16**	**357.53**	**#7,#14,#16**
15	#7,#16	365.54*	#7
16	#7	366.32	#7

*Model significantly different (p < 0.05) from the previous model, e.g., for injury occurrence model number 9 differs significantly from model number 8.

Although the majority of respondents (over 84%) did not receive any physiotherapy after sustaining the first injury, over 84% of participants declared that they recovered fully. Contrary, in the group of pole dancers who sustained a re-injury, almost 83% of respondents did not fully recover (Table 3) even though the majority of them were treated with some kind of physiotherapy (over 88%). Over 30% percent of pole dancers (among those who were injured and those who sustained a re-injury) declared that the consequences of injury always influence the current level of their activity. Moreover, 50% of both groups constantly experience pain discomfort (Table 3).

**Table 3:** Distribution of pole dancers (n,%) with different codes (yes/no or never/sometimes/always) of analysed factors.

	Injured (n = 276)			Re-injured (n = 156)		
Variable/factor	Yes n (%)	No n (%)		Yes n (%)	No n (%)	
Physiotherapy (y/n)	44 (15,9)	232 (84,1)		138 (88,5)	18 (11,5)	
Pause in training (y/n)	121 (43,8)	155 (56,2)		102 (65,4)	54 (34,6)	
Severe injury (y = > 28dys/no)	50 (18,1)	226 (81,9)		136 (87,2)	20 (12,8)	
Full recovery (y/n)	234 (84,8)	42 (15,2)		27 (17,3)	129 (82,7)	

	Never n (%)	Sometimes n (%)	Always n (%)	Never n (%)	Sometimes n (%)	Always n (%)
Current pain occurrence (y/n)	11 (4)	126 (45,7)	139 (50,4)	7 (4,5)	69 (44,2)	(51,3)
Do they influence current activity (y/n)	130 (47,1)	53 (19,2)	93 (33,7)	74 (47,4)	29 (18,6)	53 (34)

Y = yes, n = no, dys = days.

## Discussion

The results of the present study showed that only a few factors were associated with injury or re-injury occurrence in this cohort of female pole dancers. As regards the first injury, in terms of participant-related factors, the increased height of the pole dancer significantly increases the odds of reporting a history of injury. Higher volume of total months of training experience, total pole dance-specific training and higher volume of any additional training activity, categorized as training-related factors, also significantly increased the odds of injury. Apart from these explanatory variables, the best model for injury occurrence also included weight of the pole dancer, history of previous non-PD related injuries, and the role and experience of the pole dancer (Table 2). As for re-injury, the association among re-injury occurrence and the variables related to post-injury situation was confirmed. A shorter pause in training and incomplete recovery significantly increased the odds of re-injury. The only training-related factor increasing the odds of re-injury was the lower level of experience in PD training (beginner or basic level).

In our study the number of pole dancers who reported sustaining an injury (over 86%), and re-injury (over 56%) was higher than in the study of Naczk et al., where 36.7% women in the pole dancing group had been injured (some were injured more than once)^6^. These differences may result from a different observation period, because Naczk recorded injuries during the last 2 years, while in our study, subjects reported injuries throughout their careers. Anyway, the data confirms that pole dancers are prone to sustaining an injury, and tailored injury prevention strategies should be implemented to decrease these statistics.

The association between a higher number of weekly training hours and increased incidence of pole specific injuries was found previously in the study by Soini and Iaine^13^. These findings, similarly to our results, confirmed also the following tendency: the more a pole dancer participates in the training, the more frequent she is injured.

Usually, the higher the training intensity, the greater the risk of injury^6^. However, performance fluctuations might also contribute to injury risk^14^. In our study, lower level of PD experience was associated with slightly higher occurrence of re-injury. This was in agreement with the previous study of Lee and Iaine which suggested that doing higher levels of pole dancing did not result in more injuries^13^. Since the training experience improves grip strength and postural stability in pole dancers^5^, the lower number of injuries in the group of pole dancers with more experience could be linked with the ability to better perform and the higher degree of safety while dancing^6^, but these need to be verified in longitudinal, prospective studies.

Although former studies^11–13^ looked at type and localization of pole dancing injuries, and they presented very broad characteristics of PD specific injuries, no data was found on explanatory factors for sustaining re-injury in this specific population. To our knowledge, our study is the first study to analyse the effects of participant-, training- or post-injury factors on re-injury occurrence in a large cohort of female pole dancers. The calculated logistic regression model revealed three main variables associated with re-injury occurrence in our sample: lower level of training experience, shorter pause before returning to training post-injury, and subjective quantification of incomplete recovery. Participants who declared advanced or extreme level of PD skills had lower odds ratio of re-injury occurrence then beginner or basic pole dancers. There is a common problem of participation in dance sports and pole dance despite self-decelerated pain sensation^15,16^. Dancers probably ignore minimal and mild level of any kind of problems and continue normal training, regardless of the pain, trying to avoid manoeuvres that caused the pain^6^. As it is confirmed in other sports, implementation of overuse injures monitoring systems^17^, might be relevant to stress this relevant issue, and to minimize the risk of sustaining a re-injury. Post-injury management and return-to-dance progression based on time and function criteria should be present in the rehabilitation process, covering optimal time for proper reintegration into dance participation^18^. Additionally, shortening the recovery phase might be associated with re-injury occurrence, as it was confirmed not only by our study, but also by Lee and co-authors, who reported that pole dancers who had 3 to 6 years of pole sport experience, were 3.9 times more likely to require longer time of recovery, than those who practiced pole dancing for 2 years or less^11^.

What was surprising for us was that over 84% of injured pole dancers did not report any participation in physiotherapy process as the post-injury strategy. As this was unexpected, we did not ask the participants to provide further information on whether they received any treatment. It is therefore impossible to analyse the reasons thereof; it is unknown to us whether participants did not feel like they should receive any physiotherapeutic or medical consultation, or whether there was perhaps no such specialist in their vicinity, eg.in the club where they train. Lee and Iaine also noted a similarly low percentage of pole dancers who use the help of other experts^13^. They found that only 22.8% or participants were doing structured rehabilitation with a physiotherapist or a personal trainer at the gym.

Present study had some potential limitations. The cross-sectional study does not allow for establishing a cause-and-effect relation. Not all active dance schools are associated in the Polish Pole Sports Community, and thus, the sample size could be larger to provide a relevant power of study. No information was collected about any other health related problems of dancers that might influence the current level of their functional capability, e.g., history of surgical treatment. No detailed information about individual intensity and volume of training was collected, thus the calculation of a precise injury rate was impossible. No information on percentage of eligible pole dancers who did not return a survey is presented and the data can be biased due to self-report retrospective study design. Moreover, no control or comparison group was included in the study. Taking into account the development of PD as the sport during last few decades, and its specificity in physiological and physical demands, it might be necessary in the future to develop and implement the standards for data injury collection, similarly to other sports^19^. Additionally, due to the fact that the retrospective and cross-sectional study design has serious limitations^20^, further studies should rely on prospective observation, which is necessary for exhaustive establishing the extent, aetiology and mechanisms according to systematic approach in injury prevention^21,22^.

## Perspectives

Present study identified a few factors that are related with injury and re-injury occurrence in female pole dancers. Athletes with higher height and spending more time on pole-specific training in studio and on other forms of training have higher odds of sustaining an injury. Pole dancers with lower level of experience in PD training, who sustained an injury, and who had a shorter pause between the moment of injury and the return to performance, and thus who did not fully recover, have higher odds of sustaining a re-injury. Given that over 85% reported having sustained some type of injury during their career, it is worth considering how to reduce this percentage in the future. Perhaps the right solution would be to develop and implement pole dance specific preventive exercises dedicated to this group. However, verification of this assumption requires further research.

## Material and methods

The study was designed as cross-sectional survey with retrospective collection of all necessary information. Between March and June 2019, fifty pole dance schools (recognized by the Polish Pole Sports Community associated in the IPSF) from 12 out of all 16 provinces in Poland were invited to the study and its representatives were asked to distribute the online questionnaire among female pole dancers. The survey consisted of two sections with 13 and 12 questions respectively, grouped according to the following aspects: (1) demographic data and pole dancer profile including: age, height, weight, BMI, school name, previous injuries not related with pole dance); (2) PD training-specific and sport participation data related to: the level of experience, weekly participation volume, additional activities; and (3) injury history i.e. type, location, severity of injury expressed as time-loss data (confirmed by medical staff). The participants were informed that the questionnaire served for research purposes and the authors were interested in analysing the factors that are related with injury and re-injury occurrence in this specific population. All responders declared their voluntary participation in the study. The Bioethical Committee of Silesian Medical University provided ethical approval of the study (no. PCN/CBN/0022/KB/145/19). The experiments in the present study were performed in accordance with the Helsinki Declaration.

Assuming a 95% confidence level and a 5% margin of error, which is common in epidemiological research, a sample size of 317 was required, in a population of 1800 female pole dancers in Poland.

To meet the criteria for this study, a participant had to be an active pole dancer for at least 6 months, and she had to be registered in one of the official PD schools in Poland. No other inclusion or exclusion criteria were applied.

To investigate what factors are associated with an injury and re-injury occurrence in a cohort of female pole dancers we used a logistic regression with „injury occurrence” as dichotomous response variable (0 = no injuries, 1 = one or more injuries). As the explanatory variables (independent variables) we used several modifiable and non-modifiable factors. They were grouped into three categories: participant-, training-, and variables related to post-injury situation. The main participant-related explanatory variables investigated were: age in years (factor #1), weight in kilograms (factor #2), height in centimetres (factor #3) (all of these factors were qualified as continuous type of measures), abnormality of Body Mass Index of the pole dancer (factor #4), dichotomously categorized as 0 = normal or healthy weight (when BMI is ranged 18.5–24.9), and 1 = abnormal weight (underweight when BMI < 18.5 or overweight when BMI >  = 25), and information about any previous injury sustained by the pole dancer before regular participation in PD training dichotomously categorized as 0 = no and 1 = yes (factor #5). The training-related explanatory variables included the information about the role and experience of the pole dancers. They were dichotomously categorized as 0 = participant of the classes and 1 = instructor for the PD classes (factor #6), level of experience in PD training, 0 = beginner or basic level and 1 = advanced or extreme level (factor #7), information about any items used by pole dancer for her protection classified as 0 = no and 1 = yes (factor #8). Additional variables, which were considered as continuous measures were total months of training experience in pole dance, expressed in months (factor #9), pole-specific training performed in the dance studio expressed in minutes per week (factor #10), similarly calculated total volume of pole-specific training performed in the dance studio and additional physical activities, e.g. swimming, riding a bike, running (factor #11), and minutes dedicated for the warm-up before the training session (factor #12). For those pole dancers who sustained an injury, the participant- and training-related factors mentioned above were also used to assess the relative contributions for sustaining any re-injury (0 = no re-injury, 1 = re-injury reported). Additionally, 4 more explanatory variables dedicated to post-injury situation were added to the logistic regression model: dichotomous information whether the physiotherapy was applied after sustaining an injury or not, 0 = no, 1 = yes (factor #13), information whether the pole dancer had a pause from the training session or not, 0 = no, 1 = yes (factor #14), information whether the pole dancer sustained severe injury or not, 0 = no, 1 = yes (> 28 days of time loss in training) (factor #15) and information whether the pole dancer fully recovered, 0 = no, 1 = yes (factor #16). To evaluate the associations of all explanatory variables with injury and re-injury, two models of logistic regression analyses were performed and odds ratios with 95% confidence intervals (CI) were calculated. Variable selection for logistic regression models was based on backward stepwise empirical approach removing non-significant variables using p < 0.05 as the cut-off level^23^. We started with a basic model containing all the predictors and then by removing the weakest contributor in every single model we finally developed the model containing only the variables significantly relevant for the injury or re-injury occurrence. Akaike Information Criterion (AIC) was calculated to observe the difference between the models analysed in backward stepwise, and the best subset models were specified among them, based on the minimum value of AIC^24^. The calculations were performed using the PQStat 1.6.2. statistical program (PQStat Software, Poznan, Poland). A p-value of less than 0.05 was considered to be indicative of statistical significance.
